# Greener Solution to Waste Corn Stalks and Shortage of Asphalt Resource: Hydrochar Produced by Hydrothermal Carbonization as a Novel Performance Enhancer for Asphalt Binder

**DOI:** 10.3390/ma14061427

**Published:** 2021-03-15

**Authors:** Xiaoming Wu, Chichun Hu

**Affiliations:** School of Civil Engineering and Transportation, South China University of Technology, Guangzhou 510641, China; ctxmwu@mail.scut.edu.cn

**Keywords:** corn stalk, modified binder, anti-aging effect, rheological tests, chemical analysis

## Abstract

Utilization of waste corn stalks (CS) has seized extensive attention due to high annual output and hazardous impact of piling aside or direct combustion on environment. However, previously there has been a lot of emphasis on improvement of its energy efficiency as solid fuel while limited investigations are available which explore the possibility of applying corn stalks as performance enhancer in asphalt binder. The purpose of this study is to examine the potential of employing hydrochar as modifiers in asphalt binder by a series of experimental tests. In this study, two hydrochar were produced from corn stalks by a novel process called hydrothermal carbonization at a different reaction temperature. The two hydrochar and their responding hydrochar-modified asphalt (HCMA) were tested by chemical and rheological tests. Chemical analysis detected the interaction between hydrochar and binder factions, resulting in poor compatibility but satisfying anti-aging property. Even though hydrochar increased the viscosity of bitumen, implying worse workability, and caused poor storage stability, ameliorated performance of asphalt binder at high temperature by incorporating hydrochar was verified by various criteria such as higher performance grade (PG) failure temperature and lower non-recoverable creep compliance (*J*_nr_). Moreover, higher reaction temperature makes hydrochar’s particles smaller and more homogeneous, which results in slightly lower enhanced high temperature performance, more satisfying workability, better storage stability, and greater anti-aging effect of hydrochar-modified asphalt. Eventually, this study provided a promising win-win solution to environment problems concerning corn stalk treatment and shortage of asphalt binder. Further exploration of methods to improve HCMA’s storage stability, real-scale corroboration on trial section and life cycle assessment of asphalt pavement containing hydrochar modifiers will be followed in the future.

## 1. Introduction

As more attention has been drawn on sustainable development, concern of the environmental problem induced by the abundant discarded corn stalks (CS) produced from agricultural industry is growing rapidly, which is more severe in China because of its high annual output of corn stalks (about 250 million tons per year) and poor efficiency of waste utilization [1]. Traditional treatment of corn stalks is combusting them directly as a kind of fuel, whose energy efficiency is low and consequently causes large amounts of CO, CO_2_ and NO_2_ emissions leading to increase in diseases [2]. More commonly, farmers would rather stack them on the field than try to utilize them; however, this accidentally results in eutrophication, which is hazardous to farmland [3]. In other words, great benefits will be achieved if proper treatment is undertaken for corn stalks. Many efforts have been made to develop an environmentally friendly solution to waste corn stalks, consisting of hydrothermal process, pyrolysis and hydrothermal carbonization [4,5]. Not only economic but also environment-friendly processes for corn stalks are drawing extensive attention.

Flexible pavement, namely, asphalt pavement, has taken the first place to be paved in highway because of its exclusive advantages such as improved smoothness, low driving noise, and accessible maintenance. As large-scale pavement construction has been undertaken from the 21st century, especially in developing countries, demand for asphalt binder, a by-product from the petroleum industry, has consequently risen at an unbelievable speed. Similar to other unsustainable resources, there is a contradiction between the growing demand for asphalt binder, and its decreasing annual production restricted by local environmental protection policy [5]. What is more, the above contradiction leads to a higher price of asphalt binder. Great efforts have been made to cut down the consumption of asphalt binder by exploring alternative binder, as well as reducing binder content in asphalt mixtures by modification technique. For the sake of heightening mechanical properties of asphalt pavement, such as rutting resistance at high temperature, fatigue resistance at intermediate temperature, and fracture resistance at low temperature, numerous materials have been developed as modifiers, among which styrene–butadiene–styrene conjugated copolymer [6] is the most effective one for its improvement in overall performance of asphalt pavements.

In terms of biomass processes, several representative studies should be mentioned. Firstly, Dhasmana et al. employed a hydrothermal liquefaction technique to convert biomass such as spirulina algae, swine manure, and nanoalgae into bio-binder, which exhibited distinctly different rheological and chemical properties compared to petroleum binder [7]. As supplement, Fini et al. put forward that bio-binder produced from swine manure could improve asphalt binder’s low temperature performance while cutting down the construction expense of flexible pavement by partial replacement [8]. However, Yang et al. drew a conclusion that the compatibility with asphalt binder would decrease if a greater fraction of bio-binder was incorporated [9]. Moreover, Walters et al. affirmed that incorporating bio-char, acquired by a filtration procedure after bio-binder was produced, could improve asphalt rheological behavior and aging susceptibility [10]. Additionally, reduced temperature susceptibility and better rutting, cracking and moist resistance of bio-char-modified asphalt mixture were detected [11,12]. Furthermore, Zhang et al. appraised the effect of bio-char particle size on the rheological characteristics of binder and put forward that smaller-sized bio-char particles could lead to superior rutting resistance and anti-aging properties due to the porous structure and rough surface [13]. Recently, Zhou and Adhikari discovered that the flow-induced crystallization could be improved by adding bio-char [14]. Differently, Araújo et al. developed a polyester obtained from sugarcane bagasse-derived polyol through pyrolysis, which was polymerized with pyromellitic anhydride, as new additives for binder [15]. There are several studies investigating the potential of applying ashes from biomass combustion as aggregate fillers for asphalt mixtures [16,17,18], antioxidant [19] or performance enhancer [20] for asphalt binder. Specifically, Chen et al. invented a preparation method of corn stalk fiber involving sodium hydroxide [1] and evaluated the effect on binder [21].

In the past few years, biomass like corn stalk has been regarded as kind of fuel in the agricultural industry when processed. It is commonly thought that the focus is to generate highly energy-efficient renewable fuel resources using biomass. Towards this research direction, hydrothermal carbonization stands out from other thermal chemical process due to its improvement of upgrading the fuel properties [22] and dewatering of wet-basis biomass [23]. However, hydrochar produced from hydrothermal carbonization using biomass such as corn stalk seems not suitable for biofuel production [11]. On the contrary, utilizing hydrochar as binder modifiers instead of solid fuel seems more reasonable. As proof, Chen et al. made worthful achievement by exploring an innovative way of converting corn stalks to fiber material [1] and performance evaluation of asphalt modified by corn stalk fiber [21]. What is more, Bao et al. investigated the chemical composition and rheological properties of bio-asphalt, the residue from corn waste processed through acidification and polymerization [24]. In addition, Dong et al. examined the effects of bio-binder made from corn on rheological properties of asphalt binder and performance of mixture [25]. Despite some valuable studies above related to waste corn material having been conducted, limited studies seriously consider the thought of exploiting hydrochar and draw attention from stakeholders in the asphalt industry. It is of immense benefit both environmentally and economically if corn stalks are treated as associated with asphalt industry. Waste corn stalks can be converted to hydrochar, which is an innovative method to consume corn stalks. Moreover, due to the properties of hydrochar, it has great potential to be employed as asphalt modifier. Hence, the purpose of this study is to comprehensively examine the potential of employing hydrochar in asphalt binder as modifiers by a series of experimental tests. Emphasis was placed on the mechanism how reaction temperature affects the properties of hydrochar and the effect on hydrochar-modified asphalt by means of chemical analysis and rheological tests. Through a novel method called hydrothermal carbonization, waste corn stalks from the agricultural industry could be converted to hydrochar, which serves as an innovative asphalt modifier in pavement engineering. Mountains of waste corn stalks could be consumed, while performance of asphalt pavement could be improved, which would consequently cut down the enormous cost of asphalt material. It is anticipated that this study would provide a win-win solution to environment problems concerning corn stalks treatment and shortage of asphalt binder.

## 2. Materials and Methods

### 2.1. Materials and Sample Preparation

#### 2.1.1. Materials

The Pen60/70 raw bitumen obtained from Shell Co. Ltd. (Foshan, Guangdong, China), which is commonly applied in south China was selected as the base bitumen. The corn stalks which were later transformed into hydrochar were obtained from Henan province in China. Raw corn stalks were mechanically pulverized to 10 mesh and positioned in an oven at 80 °C overnight.

#### 2.1.2. Preparation of Hydrochar

**Figure 1:**
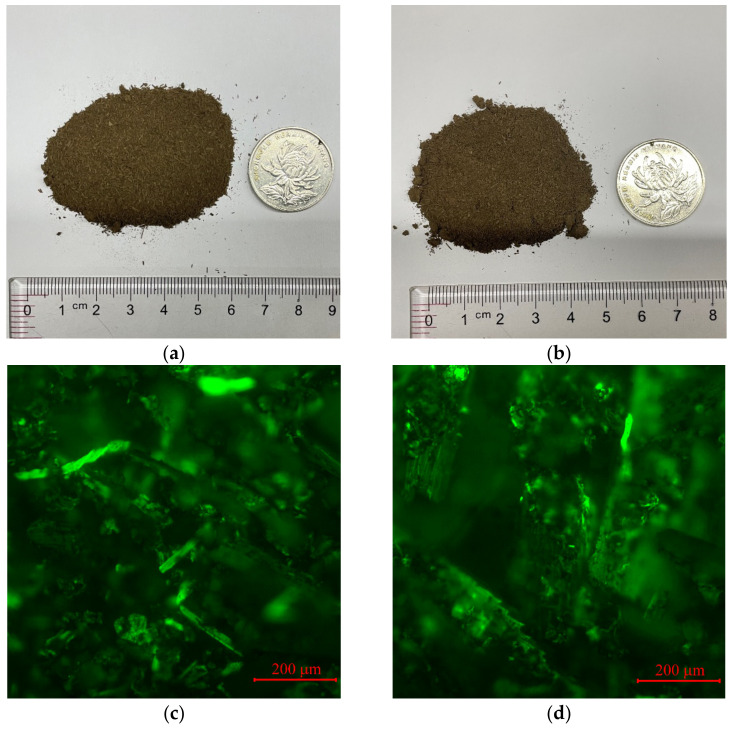
Hydrochar modifiers: (**a**) hydrochar A (HA) and (**b**) hydrochar B (HB); FM images of (**c**) HA and (**d**) HB.

For the purpose of manufacturing hydrochar, 500 g pulverized corn stalks were mixed with 7.5 L deionized water in a 10 L Hastelloy reaction vessel. The mixture was heated to 180 °C and 210 °C, respectively, for different types of hydrochar (HA, HB are abbreviations for hydrochar A, hydrochar B) and kept at this temperature for an hour. After cooling, the product was separated by sifting through filter paper. Residual moisture was removed in an oven at 80 °C. On account of different reaction temperature, HA and HB have similar physical shapes observed by eye (Figure 1a,b). Further observation of HA and HB (Figure 1c,d) on the fluorescence microscope (FM) leads to a conclusion that HA has more interconnected fiber structure which could be beneficial to the high temperature performance of modified bitumen.

#### 2.1.3. Preparation of Hydrochar-Modified Asphalt (HCMA)

**Figure 2:**
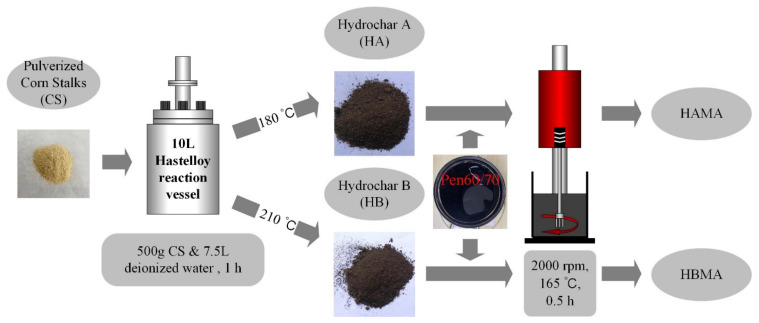
Preparation of hydrochar-modified asphalt (HCMA).

The hydrochar-modified asphalt was prepared by wet process, including manually stirring hydrochar modifiers and base bitumen with a glass rod for 5 min and then blending them with a mixer at a speed of 2000 rpm for 30 min, at (165 ± 5) °C. It was noted that swelling would happen when hydrochar was added into the hot asphalt, hence, hydrochar modifiers should be slowly poured into the base bitumen. The material dosages and mixing conditions were chosen on the basis of previous study [26]. In terms of mixing conditions, low shearing speed (2000 rpm) and shorter shearing time (30 min) are adequate for modifiers such as hydrochar which is produced from high temperature and pressure. Moreover, it consumes less energy which corresponds to the goal of environmental protection. The two prepared hydrochar-modified asphalt were called HAMA (HA-modified asphalt) and HBMA (HB-modified asphalt) for short, respectively. A batch of approximately 500 g hydrochar-modified asphalt could be manufactured after a standard preparation process. Figure 2 illustrates the preparation process of the hydrochar-modified asphalt from the stage of crushed corn stalks.

### 2.2. Testing Program

The experimental scheme of this study is summarized in Table 1.

#### 2.2.1. Penetration and Softening Point

The fundamental properties of test bitumen were appraised by two traditional tests, namely, penetration [27] and softening point tests [28]. As an index to evaluate the hardness and consistency of test bitumen, penetration is defined by the depth (0.1 mm) of a standard needle (100 ± 0.1 g) sinking into the test bitumen sample for a specified loading time (5 s), at a selected temperature (25 °C). Considered as an indicator to characterize the high temperature property of bitumen, softening point is determined by the temperature at which a steel ball (3.5 g) positioned on the top surface of a bitumen sample reaches a distance of 25.4 mm under gravity. Bitumen samples were prepared by being filled in rings and then placed in a water bath at 5 °C for at least 30 min. The temperature of the water bath was increased at a fixed rate of 5 °C/min during the softening point test.

#### 2.2.2. Rotational Viscosity

The workability of bitumen samples was evaluated by their rotational viscosity at 135 °C, 150 °C and 165 °C, respectively [29]. RV tests were conducted with a Brookfield viscometer (AMETEK Brookfield Company, Middleboro, MA, USA). After preliminary trials, 27# spindle and testing temperature (135 °C, 150 °C and 165 °C) were selected.

#### 2.2.3. Storage Stability

The heterogeneity of binder stored in the container is one of the major concerns for construction quality. Considered as a crucial parameter for pavement construction, storage stability determines the separation tendency of asphalt binder after high-temperature storage. The ASTM D 7173 [30] procedure was referred to imitate the storage condition of modified binder (being stored at high temperature). The following steps describe the experimental procedure: (1) filling an aluminum tube (with 25 mm and 140 mm length) with modified binder which was heated to reduce its viscosity in advance; (2) enclosing the tube and placing it vertically in air heater at 163 °C for (48 ± 1) h; (3) transferring the tube from the air heater to a freezer at 5 °C; and (4) segregating the tube horizontally into three uniform parts and keeping the top and bottom portions for the softening point test while the middle one was discarded. Difference in softening point was employed to evaluate the storage stability of asphalt binder.

#### 2.2.4. Temperature Sweep and Multiple Stress Creep Recovery (MSCR)

**Figure 3:**
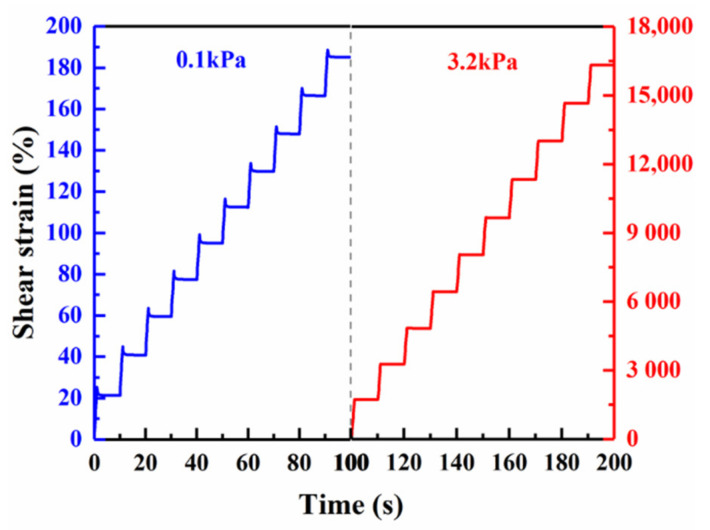
Diagram of multiple stress creep recovery (MSCR).

To determine the performance grade (PG) of test bitumen, the dynamic shear rheometer (DSR, Malvern Kinexus Lab+, Malvern Panalytical, Malvern, UK) test [31] was employed in this study. The 25 mm parallel plate geometry was selected, setting a 1 mm spacing for both the temperature sweep test and the multiple stress creep recovery (MSCR) test. Considering that the HCMA would be utilized in South China, where the annual lowest temperature rarely reaches 0 °C, only high temperature performance was investigated in this study. The temperature sweep test, from which Superpave rutting factor and failure temperature could be derived, was conducted for the sake of high temperature performance evaluation. The temperature sweeping test started from 64 °C and then the temperature would increase with a gap of 6 °C till the detected rutting factor (G*/sin δ) was below the low limit commanded in AASHTO M 320 [32], i.e., 1.0 kPa for unaged bitumen samples and 2.2 kPa for short-term-aged ones. Short-term-aged bitumen samples were prepared by rolling thin-film oven (RTFO) test according to AASHTO T 240 [33]. At first, heated to liquid state in advance, (35 ± 0.5) g of asphalt binder was poured in a glass bottle. Then the bottle was placed in the rolling thin-film oven at (163 ± 0.5) °C for 85 min. Binder after RTFO test is regarded as short-term-aged bitumen. Additionally, to simulate the repeated loading and unloading process of different vehicle loads on asphalt pavement, MSCR test was performed on the same short-term-aged bitumen sample that was previously used in the temperature sweep test, but a relaxation period of at least 1 min was required before its running, as described in AASHTO T 350 [34]. It needs to be declared that the highest temperature at which rutting factor was greater than 2.2 kPa was regarded as the initial test temperature for MSCR test, in accordance with AASHTO M 332 [35]. The bitumen samples underwent creep and recovery cycles under two different stress levels, 0.1 kPa and 3.2 kPa, respectively. A regular MSCR test consists of ten creep–recovery cycles at a stress level of 0.1 kPa and immediately another ten cycles at 3.2 kPa. A single creep–recovery cycle comprises 1 s of loading period and then 9 s of rest period. Figure 3 shows the loading path of a standard MSCR sequence. Three related parameters, namely, the average percent recovery (*R*%), non-recoverable creep compliance (*J*_nr_), and stress sensitivity parameter (*J*_nr-diff_), were recorded to appraise the high temperature property of test bitumen. Eventually, applicable traffic level for corresponding binder samples were classified.

#### 2.2.5. Frequency Sweep Test

Asphalt pavements are subjected to dynamic vehicle loads, the frequency of which would affect the viscoelastic properties of asphalt materials. The correlation between the dynamic mechanical properties of a material and test frequency at a specific temperature can be expressed in a specific function, known as the dynamic thermomechanical analysis (DMA) frequency spectrum. However, the DMA frequency spectrum whose frequency range spans multiple orders of magnitude could hardly be performed, limited by the instrument’s test range. In order to overcome the limitations and obtain a master curve [36] spanning a broad frequency domain, a frequency sweep test can be employed to measure numerous frequency spectra under various temperatures over a narrow but constant frequency domain. Then, the time–temperature superposition principle was utilized to convert them to a master curve over a broad frequency domain at reference temperature (40 °C). In this study, the frequency sweep test was undertaken on HAMA and HBMA to characterize their holistic rheological properties. The frequency sweep test, frequency of whom ranged from 0.1 to 30 Hz, was conducted from 4 to 76 °C, with an increment of 12 °C. To ensure all strain during the test is within linear viscoelastic range, a strain level of 0.5% was set based on the result from amplitude sweep prior to being undertaken on the binder samples.

#### 2.2.6. Gel Permeation Chromatography (GPC)

In order to unveil the interrelationship between hydrochar and binder fractions, the molecular weight distribution analysis was conducted through gel permeation chromatography (GPC) test [37]. An Agilent 1260 GPC was employed to categorize the components of different binders according to molecular size. Binder sample was prepared as diluent solution at a concentration of 1.0 μg/mL using tetrahydrofuran as solvent, and then was positioned into the injection module after pre-filtration through a 0.2 μm syringe filter made of poly (tetra fluoroethylene). Column calibration was finished using a polystyrene solution, at a concentration of 1 mg/mL as calibration substance, *M*_w_ (weight-average molecular weight) of which was known. A calibration curve would be derived after the column calibration, which was intended to correspond retention time to *M*_w_. Two tandem chromatographic columns (PLgel 3 μm Mixed-3 and PLgel 5 μm 103 Å) were used for molecular separation. Within the procedure of the GPC test, the binder–THF solution was drained through chromatographic columns, maintained at 30 °C and controlled to flow at a rate of 1.0 mL/min for 25 min. The constituents’ concentrations in the eluent were converted from original signal response recorded by a differential refractometer, with the calibration curve, and then the derivative GPC chromatograms were further converted into the molecular size distribution.

#### 2.2.7. Fourier-Transform Infrared Spectroscopy (FTIR)

Known as a popular and reliable chemical method, Fourier-transform infrared spectroscopy (FTIR), was employed to identify the functional groups of the test samples, and to evaluate the anti-aging effect of hydrochar on asphalt [38]. In this study, the instrument used was Nicolet IS50 (Thermo Fisher Scientific, Waltham, MA, USA). The FTIR test mainly consists of the two following steps: (1) fabricating a pellet sample (around 1 mm thick) by adding KBr powder, and (2) subsequently exposing the sample to intermediate infrared ray ranging from 4000 to 500 cm^−1^ to obtain the infrared spectroscopy (recorded in transmittance). Thirty-two scans per spectrum were conducted with a resolution of 4 cm^−1^.

## 3. Results and Discussion

### 3.1. Rheological Tests

#### 3.1.1. Basic Characteristics

**Figure 4:**
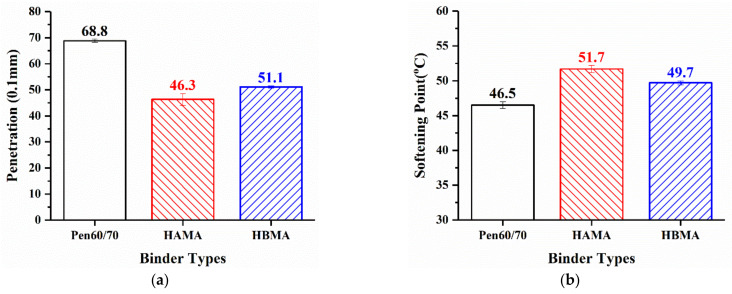
Conventional binder test results: (**a**) penetration and (**b**) softening point.

In terms of examining the basic characteristics of asphalt binder, penetration, ductility and softening point are habitually included in researchers’ laboratory plans due to accessibility. However, considering that the evenness induced by hydrochar modifiers may lead to premature brittle fracture, which consequently reduces the reliability of test results, the ductility test was excluded from the laboratory plan. Hence, only the other two tests were carried out, whose results are displayed in Figure 4. As apparent from the figure, both incorporation of HA and HB decreased the penetration value of Pen60/70, making the binder thicker. Compared to Pen60/70, the softening point values of HAMA and HBMA were mildly 5.2 and 3.2 °C higher, respectively. Figure 4 reveals that the HCMA binders generally exhibited more satisfactory performance at high service temperature. Not only that, the influence of HA on the empirical parameters was more noticeable than that of HB.

#### 3.1.2. Workability

**Figure 5:**
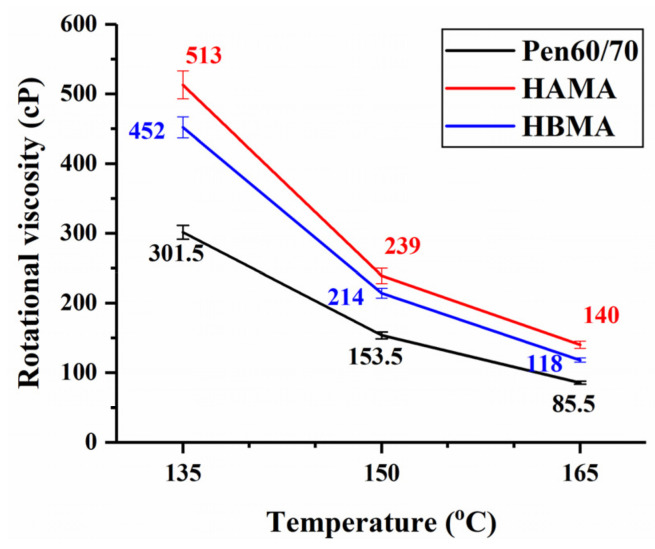
Rotational viscosity test results.

It has long been known that rotational viscosity of bitumen is a useful parameter to determinate the proper mixing and compaction temperature of asphalt mixtures. Figure 5 plots the dynamic viscosity of test binders at three different temperatures. In line with similar research [26], the addition of hydrochar modifiers slightly raised viscosity, causing an adverse impact on the workability. This phenomenon might be attributed to the stiffening effect of corn stalk fiber residues, which made the binder more viscous and consequently lowered its fluidity during mix production. The detrimental impact of HA on workability was more noticeable. As demonstrated in AASHTO specification [32], dynamic viscosity at 135 °C must not exceed 3000 cP for the purpose of guaranteeing that the asphalt mixture is properly blended and compacted at the designed void ratio. It is delightful to find out that both HAMA and HBMA conformed above requirement, indicating fairly sufficient workability of HCMA.

#### 3.1.3. Storage Stability

**Figure 6:**
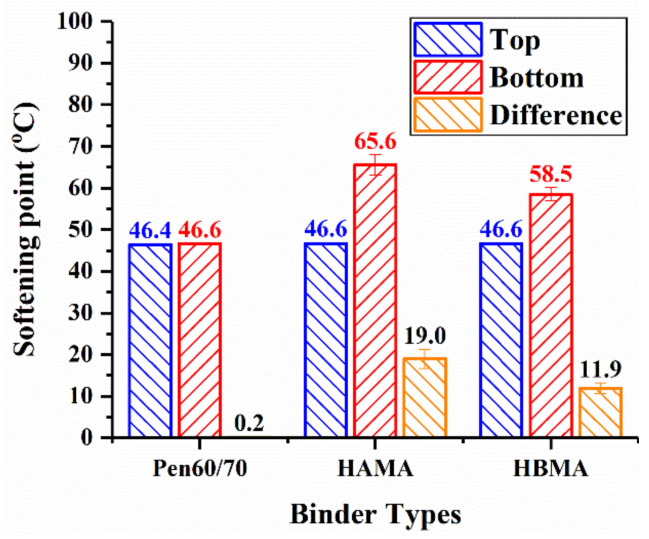
Storage stability test results.

Difference in softening point is a reliable indicator to evaluate the storage stability of asphalt binder. A requirement is specified in ASTM D 5892 [39] that the difference in softening points between the top and the bottom parts of qualified binder sample should not exceed 2.5 °C. Based on the results detailed in Figure 6, it was noted that HAMA showed a 19.0 °C softening point difference, indicating the worst storage performance, followed by HBMA with 11.85 °C while the base binder exhibited the best storage performance with only 0.2 °C. Better storage stability of HBMA could be ascribed to HB’s more homogeneous distribution in asphalt binder. Both HAMA and HBMA failed to meet the requirement specified in ASTM D 5892 [39], which meant further modification was needed to improve the storage stability of HCMA before its widespread application in realistic pavement construction.

#### 3.1.4. Rutting Resistance

**Figure 7:**
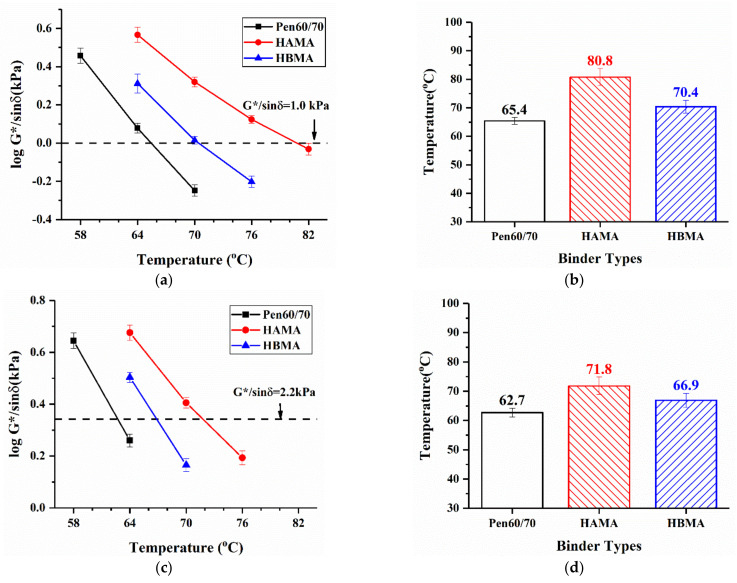
Temperature sweep test results: (**a**) rutting factor (unaged); (**b**) failure temperature (unaged); (**c**) rutting factor (short-term-aged); and (**d**) failure temperature (short-term-aged).

The high temperature performances of test binders were appraised by two DSR tests, namely the temperature sweep test and MSCR test. The correlation between G*/sin δ and test temperature is displayed in Figure 7a,c. Moreover, the test results of failure temperature, at which G*/sin δ is equivalent to respective threshold for unaged specimens and short-term-aged ones are visible in Figure 7b,d. As can be seen from Figure 7b,d, hydrochar modifiers substantially heighten the failure temperature of base asphalt in both unaged and short-term-aged levels. As depicted in AASHTO specification [32], for unaged binder, Pen60/70 accorded with the provision of PG64, while HAMA and HBMA could be identified as PG76 and PG70. Similarly, for short-term-aged binder, Pen60/70, HAMA, and HBMA were identified as PG58, PG70, and PG64, respectively. If rutting factor was chosen as the only criterion, HAMA seemed to provide better enhanced high temperature performance (a whole PG grade) compared with HBMA.

**Figure 8:**
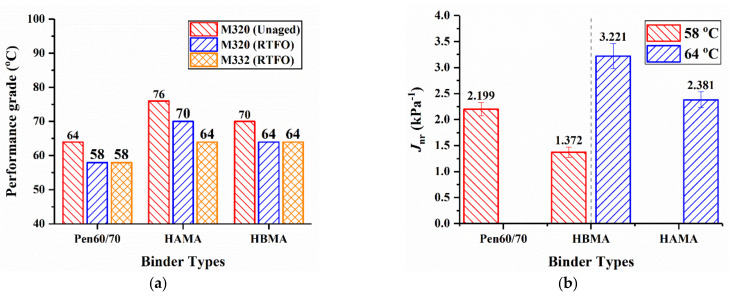
Comparison of MSCR results: (**a**) Performance grade and (**b**) *J*_nr_.

However, when the asphalt binders were further evaluated by MSCR test, the difference of performance grade between HAMA and HBMA would be narrowed. Despite rutting factor can well assess the performance of base asphalt at high temperature, the reliability is reduced when modified asphalts are appraised only by rutting factor. As stated in AASHTO M 332 [35], extra criteria, including *J*_nr_ and *J*_nr-diff_, need to be taken into consideration when performance grade is appraised in order to ensure that the test binder can exhibit good elastic behavior at standard traffic level. *J*_nr_ at 3.2 kPa cannot be greater than 4.5 kPa^−1^, for “S” grade, or 2.0 kPa^−1^, for “H” grade. The requirement of *J*_nr_ might lower the PG of test binder, which is presented in Figure 8a. The non-recoverable creep compliance (*J*_nr_), recovery ratio (*R*%) and corresponding traffic level from the MSCR test are given in Table 2. It is remarkable that the *J*_nr_ of HAMA exceeds 4.5 kPa^−1^ at 70 °C and meets the requirement at 64 °C, hence, it should be identified as PG64 instead of PG70. Similarly, Pen60/70 and HBMA should be identified as PG58 and PG64, respectively. Both short-term-aged HAMA and HBMA achieve the same PG using MSCR test, which might be more reasonable. As detailed in Figure 8b, the unmodified bitumen displayed the higher *J*_nr_ value than that of HBMA at both stress levels at 58 °C. Compared with Pen60/70 at 58 °C, HBMA showed lower *J*_nr_ values, implying heightened rutting resistance, which cohered with previous test results in this study, softening point and Superpave rutting factor. Additionally, HAMA had smaller *J*_nr_ values than that of HBMA at 64 °C, which meant that enhanced rutting resistance was promoted by HA. The above inferences were consistent with their responding traffic level. Similarly, as detailed in Table 2, comparison of the *R*% results (Pen60/70 verse HBMA at 58 °C and HAMA verse HBMA at 64 °C) verified that HCMA recovered more than base asphalt did within the rest period. The greater recovery ratio of HCMA supported the viewpoint that the addition of hydrochar leads to superior elastic behavior of asphalt, more deformations of which can be recovered.

#### 3.1.5. Overall Rheological Behavior

As elaborated in Section 2.2.5, the frequency sweep of binder sample was performed at various temperatures and frequencies. According to the time–temperature superposition principle, the procedure of developing the master curves of complex shear modulus G* at 40 °C was expatiated by the following steps: Firstly, second-order polynomial form of the shift factor relationship (Equation (1)) was substituted into the general form of sigmoidal function (Equation (2) and then derived the final form Equation (3)); Secondly, nonlinear fitting, which consisted of 3000 iterations, was performed with Equation (3), to determine the six constants (*δ*, *α*, *β*, *γ a*_1_, *a*_2_). Regression constants of the sigmoidal function and second-order polynomial are listed in Table 3.

(1) $$\log f_{r}=\log f+a_{1}(T_{R}-T)+a_{2}{\left(T_{R}-T\right)}^{2}$$

where *f_r_* refers to the reduced frequency at the reference temperature *T_R_*, *a*_1_ and *a*_2_ correspond to model constants, while *f* stands for the loading frequency at the test temperature *T*.

(2) $$\log(G^{*})=\delta +\frac{\alpha }{1+e^{\beta +\gamma \log(f_{r})}}$$

where *β*, *γ* work as coefficients describing shape of the sigmoidal function, *α* and *δ* are respectively the upper and low limit of complex shear modulus G*.

(3) $$\log(G^{*})=\delta +\frac{\alpha }{1+e^{\beta +\gamma [\log f+a_{1}(T_{R}-T)+a_{2}{\left(T_{R}-T\right)}^{2}]}}$$

**Figure 9:**
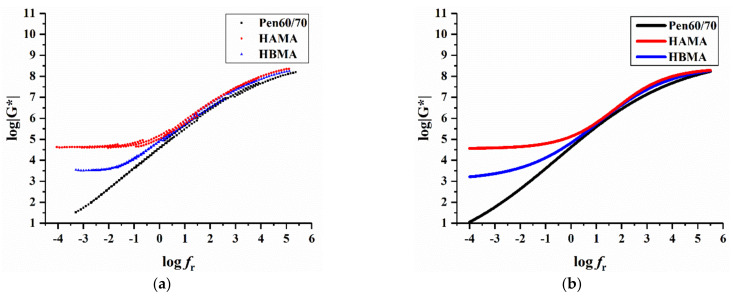
Master curves of test binders at 40 °C: (**a**) scatters of transformed result data and (**b**) regression curves of sigmoidal model.

Figure 9 plots the fitting result of investigated binders. Figure 9a is the scatters of transformed result data and Figure 9b is the regression curves of sigmoidal model. As the above-mentioned principle of viscoelastic materials describes, modulus at low frequencies is equivalent to that at high temperatures and vice versa. It is also visible that the bitumen modified by hydrochar have evidently higher complex shear modulus than Pen60/70 at low frequencies, implying superior deformation resistance. At high frequencies, all binders had similar modulus, which meant neglectable impact of hydrochar on the low temperature characteristic of base bitumen. The above inferences analyzing master curves were in good agreement with results of MSCR tests.

### 3.2. Chemical Analysis

#### 3.2.1. Molecular Weight Distribution

**Figure 10:**
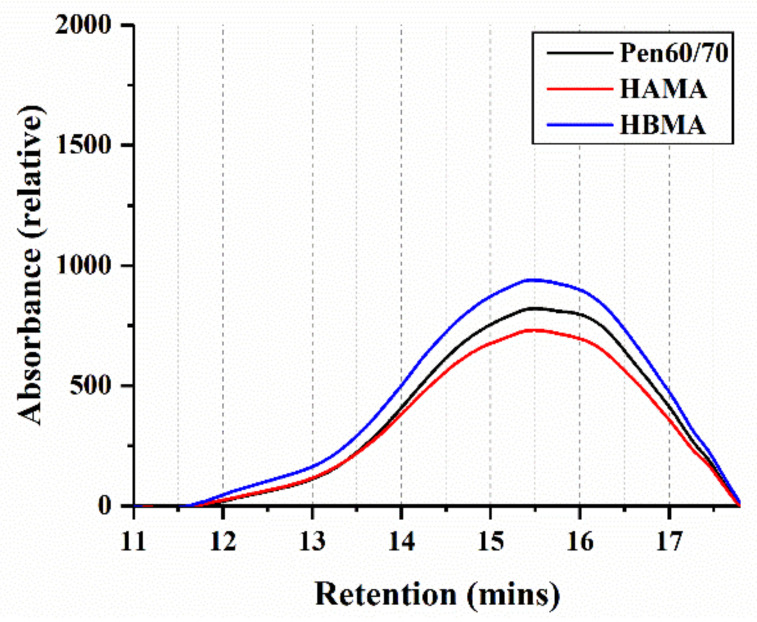
Gel permeation chromatography (GPC) chromatograms of test binders.

Figure 10 presents the GPC chromatograms of the investigated binders. Referring to related studies concerning GPC analyzation, the components of bitumen can be discriminated as different groups by weight-average molecular weight (*M*_w_) [37]. The chromatograms of target material are mainly concentrated on retention time from 11.0 to 17.8 min are presented in Figure 10, corresponding to *M*_w_ ranging from 18,647 to 134 based on the calibration curve mentioned in Section 2.2.6. Apparently, major peaks of all binders appear around 15.5 min, and no additional peak is noticeable from neither the HAMA nor HBMA, which might be attributed to the low solubility of hydrochar or reaction products in THF. Those insoluble constituents were segregated by filtration, which meant they were not taken into account in the GPC analysis. Logically, the result obtained by GPC might have slightly less significance for molecular weight distribution analysis of HCMA, because the dissolved portion of HCMA could hardly represent the entirety. After all, some information still can be obtained by the chromatograms.

**Figure 11:**
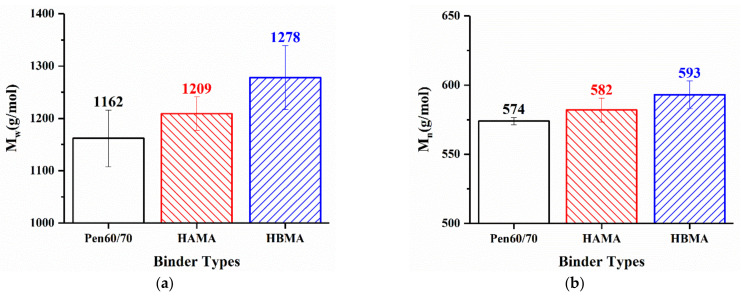
GPC result analysis: (**a**) weight-average molecular weight and (**b**) number-average molecular weight.

Table 4 lists the results of the GPC test obtained by applying signal processing and definite integral analysis. In order to depict the molecular weight distribution of investigated binders, five indicators were chosen including the peak molecular weight (*M*_p_), number-average molecular weight (*M*_n_), weight-average molecular weight (*M*_w_), z-average molecular weight (*M*_z_) and dispersity (*Đ* = *M*_w_/*M*_n_). As observed in Figure 11a,b, it is recognized that both molecular weight (*M*_w_, *M*_n_) of HAMA and HBMA are apparently higher, compared with that of Pen 60/70. This difference could be attributed to the solubility of the hydrochar fraction in THF. The reaction products between hydrochar and base bitumen probably dissolve in THF, which increases the molecular weight.

#### 3.2.2. Fourier-Transform Infrared Spectroscopy

**Figure 12:**
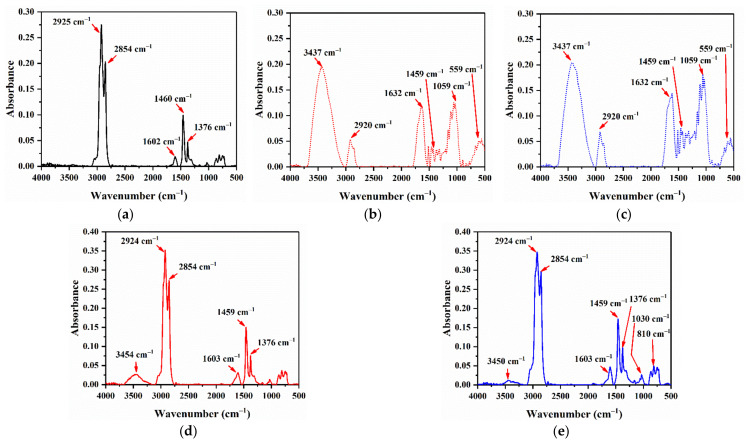
Fourier-transform infrared spectroscopy (FTIR) test results: (**a**) base asphalt; (**b**) HA; (**c**) HB; (**d**) HAMA; and (**e**) HBMA.

The generation of free radicals contributes most to asphalt aging. In other words, if the generation of free radicals can be inhibited or delayed, the aging process of binder would be remarkably hindered, which will consequently make contribution to the performance and longevity of asphalt pavement. Figure 12a–c exhibits the FTIR test result of base asphalt, HA and HB, separately. In Figure 12a, the bands at 2925 cm^−1^ can originate from asymmetric C–H stretching vibration of methyl and methylene groups, while those at 2854 cm^−1^ from symmetric C–H stretching vibration of methyl and methylene groups. As presented in Figure 12b,c, for both HA and HB, the major absorbance peaks at the 3437, 1632, 1459 and 1059 cm^−1^ are conspicuous. The absorbance band near 3437 cm^−1^ is concerned with the N–H or O–H stretching vibration of the amino groups and hydrogen-bonded hydroxyl groups, as that near 1632 cm^−1^ might be attributed to the C=O stretching vibration of carbonyl groups. Besides, peaks around 1450 cm^−1^ might be related to -CH2- scissor vibration of methylene groups, as those around 1059 cm^−1^ are induced by the stretching of C–O and S=O. As can be clearly seen in Figure 12a,d,e, the functional group compositions of HAMA and HBMA are comparable to Pen 60/70, except some peak areas being slightly different. As shown in Figure 12b–e, the peaks of HAMA and HBMA near 3437 cm^−1^ are significant compared to HA and HB, probably due to chemical reaction between hydrochar and bitumen faction. It is distinct that HAMA and HBMA have analogous major bands, but the peak areas differ greatly around 1030 cm^−1^ and 810 cm^−1^. This distinction may be ascribed to the discrepancies in the extent of dissolution and reaction of HA and HB in bitumen fractions.

**Figure 13:**
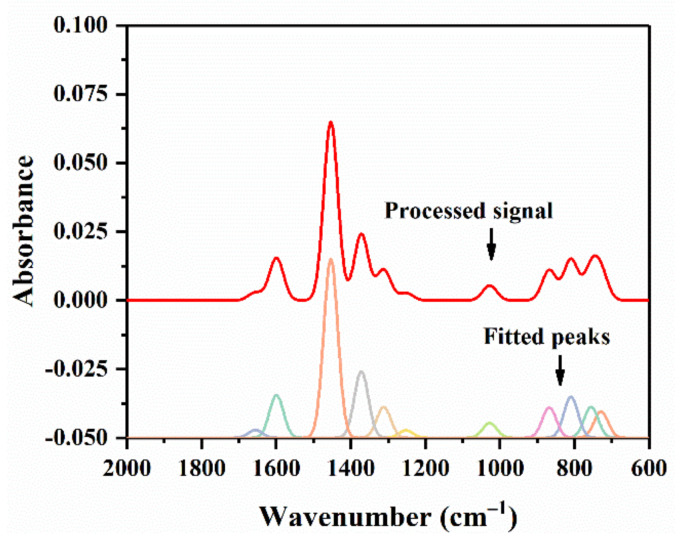
Curve-fitting analysis of FTIR for quantitative evaluation.

Curve-fitting analysis is a very effective method for competently separating FTIR overlapping peaks to obtain each absorption peak area, which improves the accuracy of quantitative evaluation. The software PeakFit (V4.12, Systat Software, San Jose, CA, USA) was employed to perform the numerical fitting. Before fitting, FTIR spectra was smoothed by signal processing. Figure 13 illustrates the process of smoothed signal and fitted peak curves. In accordance with previous studies [38] applying this method, carbonyl index *I*_C = O_ was calculated for unaged binder samples and RTFO aged ones, while derived index ratio of aging (*R*_A_) was also recorded, as presented in Table 5. *I*_C=O_ and *R*_A_ were obtained by Equations (4) and (5), respectively.

(4) $$I_{C=O}=\frac{{\mathrm{Area}\;\mathrm{the}\;\mathrm{of}\;\mathrm{carbonyl}\;\mathrm{peak}\;\mathrm{centered}\;\mathrm{around}\;1700\;\mathrm{cm}}^{-1}}{\sum {\mathrm{Area}\;\mathrm{of}\;\mathrm{the}\;\mathrm{spectral}\;\mathrm{fitted}\;\mathrm{peaks}\;\mathrm{between}\;2000\;\mathrm{and}\;600\;\mathrm{cm}}^{-1}\;}$$

(5) $$R_{A}=\frac{I_{C=O\;}\mathrm{of}\;\mathrm{RFTO}\;\mathrm{binder}}{I_{C=O\;}\mathrm{of}\;\mathrm{unaged}\;\mathrm{binder}}$$

It can be seen that both HAMA and HBMA have bigger initial *I*_C=O_ than base asphalt due to the incorporation of hydrochar modifiers, which contains carbonyl groups. However, after short-term aging, HAMA and HBMA show lower *R*_A_, which means hydrochar modifiers might prevent the oxidation of asphalt binder. Assumption was made that there would be steric hindrance phenol component in hydrochar preventing reaction between free radicals and oxygen, according to related study about lignocellulosic biomass [40]. Based on the hypothesis, more steric hindrance phenol component in HBMA than that in HAMA could be the explanation of why HBMA has a better anti-aging effect. Further confirmation of steric hindrance phenol in hydrochar should be followed. Inference could be drawn that hydrochar modifiers had convincing competence to impede the aging of asphalt binder.

## 4. Conclusions

This study presented a comprehensive investigation to explore the potential of utilizing hydrochar, a sort of innovative material produced from corn stalks as an environment-friendly performance improver for asphalt binder. An experimental plan consisting of rheological and chemical tests was carried out on hydrochar and asphalt binder modified with hydrochar modifiers, as well. The following discoveries can be acquired based on the limited test results:

1. Two kinds of hydrochar (HA, HB) were produced by an innovative method called hydrothermal carbonization. The process involves accessible reaction material, corn stalks and water, without producing hazardous by-products, which is very simple but efficient. Thanks to the above advantages, it is of great possibility that the process can be implemented on an industrial scale.
2. Interrelationship between hydrochar and asphalt binder is discovered. The tiny fiber structures of hydrochar work as a kind of reinforced skeletons within the asphalt binder, which intensify the elastic behavior, the essence of mechanical performance.
3. Hydrochar inevitably has a detrimental impact on the workability of bitumen. This phenomenon might be attributed to the stiffening effect of corn stalk fiber residues, which made the binder more viscous and consequently lower its fluidity during mix production. After all, hydrochar-modified asphalt satisfies the viscosity requirement for construction.
4. HCMA has unsatisfying storage stability exceeding the requirement in ASTM D 5892, which reveals the heterogeneous distribution of hydrochar in asphalt binder during storing.
5. The integration of hydrochar and asphalt binder presents improved performance of asphalt binder at high temperatures, which can be proved by a great deal of criteria including lower penetration, higher softening point, bigger rutting factor, lower non-recoverable creep compliance, etc.
6. The better anti-aging effect contributed by Hydrochar modifiers is observed clearly using quantitative analysis on the FTIR spectra of unaged and RTFO-aged binders.
7. Higher reaction temperatures make hydrochar’s particles smaller and more homogeneous, which results in slightly lower enhanced high temperature performance, more satisfying workability, better storage stability, and a greater anti-aging effect of hydrochar-modified asphalt.

Overall, this study ascertains the significant profit of employing corn-stalk-based hydrochar as an eco-friendly performance enhancer for petroleum binder in the pavement industry. After investigation, hydrothermal carbonization was recognized as an advisable solution for waste corn stalks for its environmental and economic benefits. Further exploration of methods, such as addition of bio-based reinforcing filler, to improve HACA’s storage stability is urgent. Then, future study including real-scale corroboration on trial section and life cycle assessment of asphalt pavement with hydrochar modifiers will follow.

## Figures and Tables

**Table 1:** Experimental scheme.

Performance	Tests	Simulated Condition	Standards
Basic characteristics	Penetration	unaged	AASHTO T 49
	Softening point		AASHTO T 53
Workability	Rotational viscosity	unaged	AASHTO T 316
Separation tendency	Storage stability	storage	ASTM D 5892, D 7173
High temperature performance	Temperature sweep	unaged, short-term-aged	AASHTO M 320
	MSCR ^a^	short-term-aged	AASHTO T 350, M332
Overall rheological behavior	Frequency sweep	unaged	N/A
Molecular weight distribution	Gel Permeation Chromatography (GPC)	unaged	N/A
Interaction identification	Fourier-Transform Infrared Spectroscopy (FTIR)	unaged	N/A
Anti-aging evaluation		unaged, short-term-aged	N/A

^a^ MSCR, multiple stress creep recovery.

**Table 2:** Multiple stress creep recovery (MSCR) test results.

Binder Types	Temperature (°C)	*J*_nr_			Recovery (%)		Traffic Level
		0.1 kPa	3.2 kPa	*J*_nr-diff_ (%)	0.1 kPa	3.2 kPa	
Pen60/70	58	2.039	2.199	7.8	0.6	−0.5	“S”
HAMA	64	1.881	2.381	26.6	8.7	1	“S”
	70	1.771	5.034	184.3	17.9	−0.5	Failed ^a^
HBMA	58	1.207	1.372	13.7	6.3	0	“H”
	64	2.892	3.221	11.4	3.4	−0.3	“S”

^a^ “Failed” means the responding binder wasn’t qualified as any traffic level at responding temperature.

**Table 3:** Regression constants of the second-order polynomial and sigmoidal function.

Constants		Pen60/70	HAMA	HBMA
Second-order polynomial	*a*_1_	0.087129	0.0937799	0.0834853
	*a*_2_	0.000585222	0.000197275	0.000490108
Sigmoidal Function	*δ*	−0.818071	4.55092	3.04264
	*α*	9.78038	3.8313	5.47337
	*β*	−0.225198	1.72356	0.725259
	*γ*	−0.416557	−0.977483	−0.684152
	R^2^@|G*|	0.999342	0.997296	0.99783

**Table 4:** Molecular weight distribution.

Binder Types	*M*_p_ (g/mol)	*M*_n_ (g/mol)	*M*_w_ (g/mol)	*M*_z_ (g/mol)	*Đ* (-)
Pen60/70	641 ± 19	574 ± 3	1162 ± 54	2699 ± 440	2.0250 ± 0.0960
HAMA	698 ± 17	582 ± 9	1209 ± 32	2809 ± 212	2.0777 ± 0.0281
HBMA	729 ± 16	593 ± 10	1278 ± 61	3130 ± 208	2.1527 ± 0.0688

**Table 5:** Result of FTIR quantitative evaluation.

Binder Types	*I*_C=O_		*R*_A_
	Unaged Binder	RFTO Binder	
Pen60/70	0.0364	0.0816	2.242
HAMA	0.0510	0.1013	1.986
HBMA	0.0586	0.0917	1.565

## Data Availability

The data used to support the findings of this study are available from the corresponding author upon request.
